# Supplementary material for: Genetic comparisons of interleukin-17 reveal a framework for complex signaling evolution
Source: bioRxiv. 2026 Apr 14:2026.04.13.718218. Preprint. [Version 1] doi: 10.64898/2026.04.13.718218 (PMC13105070; doi:10.64898/2026.04.13.718218)
Supplement: Supplement 3 [file media-3.zip › Supplemental files for paper/ERC/IL17xIL17_comparisons/IL17RE_IL17C.pdf]

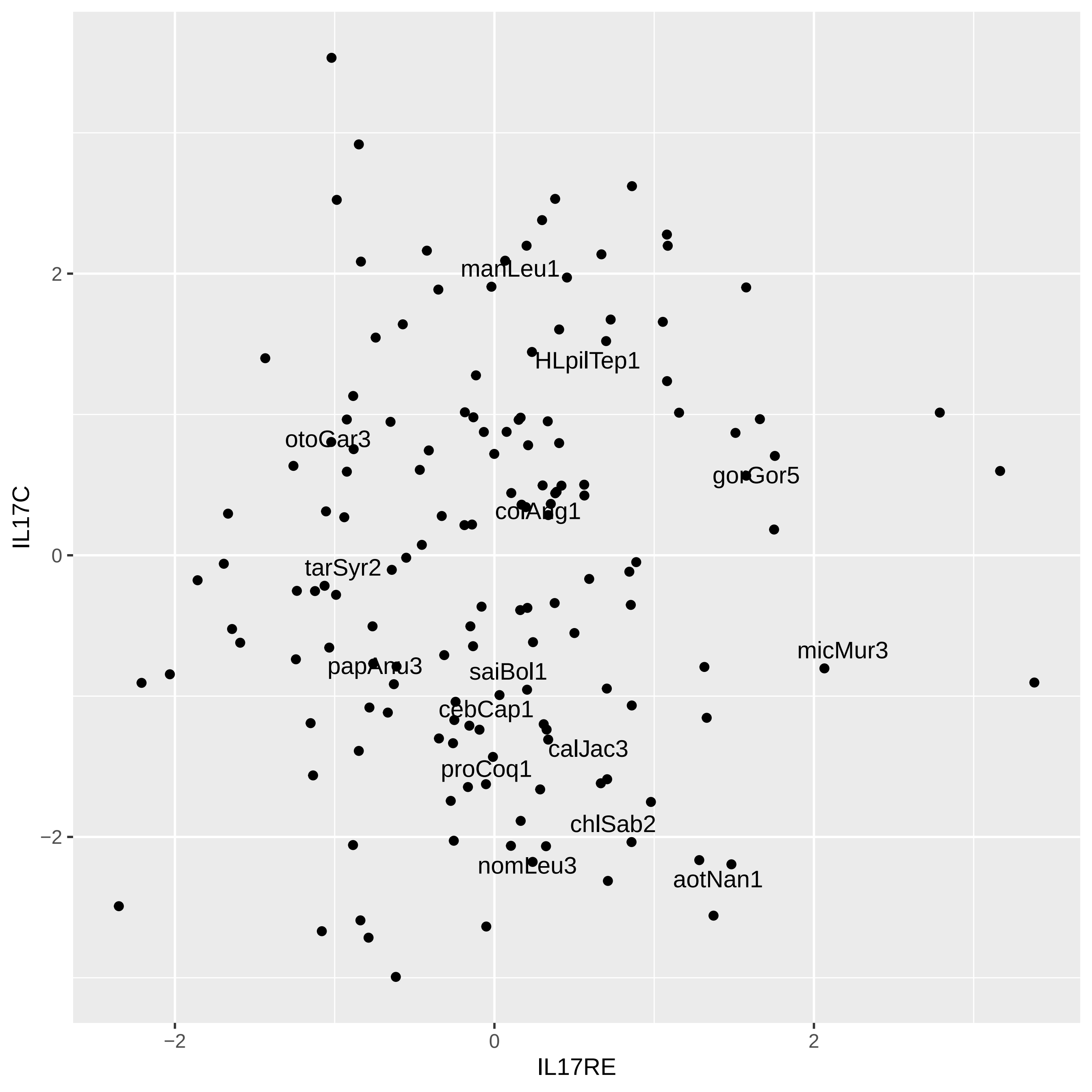
manLeu1
HLpilTep1
otoGar3
gorGor5
colAng1
tarSyr2
micMur3
papAnu3
saiBol1
cebCap1
calJac3
proCoq1
chlSab2
nomLeu3
aotNan1
IL17C
IL17RE
2
0
−2
−2
0
2
